# Tailoring of magnetism & electron transport of manganate thin films by controlling the Mn–O–Mn bond angles via strain engineering

**DOI:** 10.1038/s41598-024-53722-9

**Published:** 2024-02-08

**Authors:** P. Henning, R. Gruhl, U. Ross, V. Roddatis, V. Bruchmann-Bamberg, K. P. Stroh, M. Seibt, P. Gegenwart, V. Moshnyaga

**Affiliations:** 1 Erstes Physikalisches Institut, Georg-August-Universität Göttingen, Friedrich-Hund-Platz 1, 37077 Göttingen, Germany; 2 Experimentalphysik VI, Center for Electronic Correlations and Magnetism, University of Augsburg, 86159 Augsburg, Germany; 3 4th Institute of Physics – Solids and Nanostructures, Georg-August-Universität Göttingen, Friedrich-Hund-Platz 1, 37077 Göttingen, Germany; 4 GFZ German Research Centre for Geosciences, Helmholtz Centre Potsdam, Telegrafenberg, 14473 Potsdam, Germany

**Keywords:** Materials science, Physics

## Abstract

Strain engineering beyond substrate limitation of colossal magnetoresistant thin (La_0.6_Pr_0.4_)_0.7_Ca_0.3_MnO_3_ (LPCMO) films on LaAlO_3_-buffered SrTiO_3_ (LAO/STO) substrates has been demonstrated using metalorganic aerosol deposition technique. By growing partially relaxed 7–27 nm thick heteroepitaxial LAO buffer layers on STO a perfect lattice matching to the LPCMO has been achieved. As a result, strain-free heteroepitaxial 10–20 nm thick LPCMO/LAO/STO films with bulk-like ferromagnetic metallic ground state were obtained. Without buffer the coherently strained thin LPCMO/STO and LPCMO/LAO films were insulating and weakly magnetic. The reason for the optimized magnetotransport in strain-free LPCMO films was found to be a large octahedral Mn–O–Mn bond angle φ_OOR_ ~ 166–168° as compared to the significantly smaller one of φ_OOR_ ~ 152–156° determined for the tensile (LPCMO/STO) and compressively (LPCMO/LAO) strained films.

## Introduction

Heteroepitaxial growth of complex oxide films with perovskite structure, i.e., cuprates, manganites, cobaltates, nickelates, etc., has been considered to be a challenging task because of two important issues. *First*, the practically unavoidable lattice misfit between the film and substrate strongly limits the choice for a suitable substrate and leads to a biaxial “epitaxy stress”. The latter is added to the intrinsic chemical pressure solely determined by the cation stoichiometry and tolerance factor^1,2^ of, e.g., optimally doped (La_1−y_Pr_y_)_0.7_Ca_0.3_MnO_3_ (LPCMO)^3^. *Second*, due to the inherent coupling of electron, spin and lattice degrees of freedom^4–6^ the electronic properties of the heteroepitaxial films are strongly influenced by epitaxy stress. This usually leads to a degradation of the electronic properties of thin manganite films.^7,8^ It can be theorized that thin heteroepitaxial films of LPCMO, which is known as a prototypic colossal magnetoresistive^9^ manganite, are particularly sensitive to strain effects. Being a bandwidth- controlled manganite with increased orthorhombic distortion^10^ and enhanced electron–phonon coupling^11^, the LPCMO properties are easily influenced by lattice strain and disorder. Thus, to investigate intrinsic electron–phonon and spin-phonon interactions, e.g. controlling ultrafast reflectivity and magnetization dynamics^12^ as well as thermal conductivity^13^, stress-free thin heteroepitaxial LPCMO films are necessary.

*Stress-free heteroepitaxial manganite films* can be obtained by a trivial increase of their thickness to exceed the critical thickness which usually amounts to d_c_ ~ 60–150 nm^14,15^ and depends strongly on the film/substrate lattice misfit. Remarkably, strain relaxation in some cases has been found to be incomplete even for very thick films, d ~ 300 nm >  > d_c_^15^. This often involves the formation of oxygen vacancies^16^ as well as changes in the cation stoichiometry, both degrading the film properties. Another possibility is provided by the so called *domain matching epitaxy*^17^ known to take place for extremely large values of film/substrate lattice misfit ε > 7–8%, which is the case for e.g. manganite films grown on MgO substrates^18,19^. The stress relaxation occurs via introduction of misfit dislocations at the film/substrate interface. However, in this case the bulk-like electromagnetic properties, e.g., ferromagnetism and metal–insulator transition for optimally doped LPCMO and La_0.7_Ca_0.3_MnO_3_ (LCMO) films, can also only be observed for relatively thick films with d ≈ 100 nm^18,19^. The magnetotransport properties of thinner and seemingly relaxed LPCMO/MgO films with d = 10–20 nm, are strongly influenced by residual lattice strain and cation disorder, induced by misfit dislocations. As a result, such optimally doped films reveal a strongly suppressed magnetism in addition to insulating behavior.

The established strong strain-driven effects have also sparked the idea to employ *strain engineering* as a tool for tuning the functional properties of perovskite films.^20,21^ In principle, this can be achieved through the use of suitable buffer layers, which also need to be obtained in the relaxed state. Namely, they must be thick enough d > 100 nm to enable a perfect lattice matching as was shown earlier for lightly doped La_2_CuO_4_^22^, high-T_C_ superconducting La_2−x_Sr_x_CuO_4_/SrLaAlO_4_/SrTiO_3_ (STO)^23^ and GdBa_2_Cu_3_O_7−x_/La_0.7_Sr_0.3_MnO_3_/LaAlO_3_^24^ films. This approach appears generically similar to the search for a suitable substrate, the choice of which, however, is very limited for an arbitrary chosen film composition. Even for almost perfect lattice matching, e.g., for LCMO films grown on NdGaO_3_ (NGO) substrate, the octahedral coupling between the film and the substrate, having different octahedral tilt and rotation patterns, was shown to strongly influence the structure and properties of LCMO/NGO heteroepitaxial films^25–27^. Furthermore, the octahedral tilt/rotation angle φ_OOR_ is a well-known structural parameter controlling the structure and electronic properties of perovskite manganites^28^. It has also been considered as an important target parameter for the strain engineering of thin perovskite manganite films in order to optimize their properties^29–32^.

Recently, a fundamentally different approach, namely, the strain engineering beyond substrate limitations^33–36^, has been suggested in order to optimize structure and electronic properties of heteroepitaxial films. It is based on thickness tuning of a heteroepitaxially grown and partially relaxed buffer layer to achieve a controllable strain state of the subsequently grown complex oxide film. For example, by using a Sr_3_Al_2_O_6_ (SAO) buffer layer with the thickness of d_SAO_ ≤ 5 nm grown on STO(100) substrate, the strain in a Nd_0.5_Sr_0.5_MnO_3_ (NSMO) can be controlled up to ≈ 1% by gradual strain relaxation of the SAO buffer^34^. However, this attempt, although resulting in the relaxed NSMO film, unexpectedly, did not lead to the recovery of the intrinsically complex magnetic, electric and structural NSMO bulk behavior. Namely, the coupled ferromagnetic/anti-ferromagnetic-charge ordered and structural phase transitions around 150 K, both inherent to the bulk NSMO^37^, have not been detected in the strain-free NSMO/SLAO/STO film. A promising approach to strain engineering beyond substrate limitations has been reported by Deng et al.^35^ They demonstrated that the insertion of a Ca_0.96_Ce_0.04_MnO_3_ (CCMO) buffer layer with a thickness d_CCMO_ = 2–9 nm between a BiFeO_3_ (BFO) film and the LAO, LSAT, STO, and NdScO_3_ substrates allows one to create the desired strain state in the BFO film. As a result, a metastable tetragonal-like phase in BFO films as well as the rhombohedral-tetragonal phase transition have been demonstrated. A room temperature electrical switching in BFO-based heterostructures was realized. Finally, by using a CaMnO_3_ (CMO) buffer layer with d_CMO_ = 10 nm grown on STO(100), c-axis oriented Ruddlesden-Popper Pr_0.5_Ca_1.5_MnO_4_ (RP-PCMO) films with bulk structure and high charge order transition temperature T_CO_ ≈ 320 K were obtained^36^. A partially relaxed CMO buffer enables the bridging of a very large RP-PCMO/STO lattice misfit of about 3%. Note, that without CMO buffer the RP-PCMO films did not show c-axis orientation but rather an a-axis orientation.

Here we report the successful strain engineering of (La_0.6_Pr_0.4_)_0.7_Ca_0.3_MnO_3_ (LPCMO) films grown on STO(100) substrates by using ultrathin LAO buffer layers. Cubic STO with lattice constant a = 0.3905 nm is a standard substrate for manganite films. The LAO/STO epitaxy pair is a prominent 2DEG system^38^, for which very thin films of rhombohedral LAO with pseudocubic lattice parameter a_LAO_ = 0.379 nm and high structural quality are known to grow epitaxially on STO. Considering a large lattice LAO/STO misfit of 3% one can suppose a partial strain relaxation and, thus, an in-plane LAO lattice constant approaching that of LPCMO (a = 0.385–0.386 nm^19^) already for a very thin LAO buffer. Indeed, by tuning the LAO thickness in the range of d_LAO_ = 3–10 nm, very thin d ≈ 10–20 nm and stress-free heteroepitaxial LPCMO/LAO/STO(100) films with a bulk-like magnetotransport have been prepared. In contrast, the ferromagnetic metallic behavior was suppressed in coherently tensile strained LPCMO/STO(100) films. Furthermore, we show that the structural mechanism behind the strain-induced degradation in thin LPCMO/STO(100) films is a tetragonal distortion, accompanied by a strong decrease of octahedral tilt/rotation Mn–O–Mn angle down to φ_OOR_ ≈ 156°. Meanwhile, the strain-free LPCMO/LAO/STO film possess a much larger φ_OOR_ ≈ 166°, which favors the ferromagnetic metallic ground state of LPCMO.

## Experimental details

LPCMO and LAO films have been prepared by means of metalorganic aerosol deposition (MAD) technique^19,39^, based on spraying precursor solutions, i.e., La-, Pr-, Ca-, Mn-, and La- and Al-acetylacetonates in dimetylformamide, onto a heated substrate. Precursors taken in empirically determined molar ratios, e.g. (La + Pr + Ca)/Mn = 1.35 and La/Al = 1.2 provide the required film stoichiometry. This was further evidenced by an almost perfect surface morphology and microstructure of the films addressed in details below. STO(100), LAO(100) and MgO(200) substrates were purchased from Crystal GmbH; the STO substrates were TiO_2_-terminated^39^ prior to deposition. The films have been grown in ambient atmosphere (pO_2_ = 0.2 bar) at a substrate temperature T_sub_ = 900–950 °C under growth rate of v ≈ 0.3 nm/s. After deposition the films were cooled down to room temperature withiin 15 min.

Structure and thickness of the prepared films were characterized by X-ray diffraction (XRD) in Θ-2Θ Bragg–Brentano geometry and by small angle X-ray reflection (XRR) with Cu–K_α_ radiation using a Bruker “D8” diffractometer. In-plane epitaxy was characterized by reciprocal space mapping measured using a “Malvern Panalytical Empyrean” diffractometer. Surface morphology of the films has been visualized by atomic force microcopy (AFM) from “Bruker Innova”. Scanning Transmission Electron microscopy (STEM) was performed using a “Thermo Fisher Scientific” (TFS) Themis Z 80-300 (S)TEM operated at 300 kV, and equipped with a TFS SuperX Energy Dispersive X-ray (EDX) detector, and a Gatan Imaging Filter (GIF) Continuum 1065. The microscope was tuned for a sub-Angstrom resolution with a beam convergence angle of 21.4 mrad. Specimens for STEM were prepared with a lift-out Focused Ion Beam technique using a TFS Helios G4UC dual beam instrument. TEM lamellas were prepared along the [100] and [110] directions using a TFS Helios UC focused ion beam instrument with a beam energy of 30 kV; a final cleaning step was carried out at low energy (2 kV). The magnetic and electric properties were measured using a SQUID magnetometer (MPMS XL) and a Physical Property Measurements System (PPMS), respectively, from “Quantum Design”.

## Results

As a first step, magnetism and electron transport of thin (d = 16–20 nm) LPCMO films, heteroepitaxially grown on LAO, STO and MgO substrates by MAD technique, were studied. The films on STO and LAO show atomically smooth AFM surface morphology (see Supplemental Material^41^, Fig. SM-1), evidencing the layer-by-layer (step-flow) growth mode; the estimated mean square roughness was RMS = 0.2–0.4 nm. According to the XRD data (see Fig. SM-2a,b, ref.^40^) the LPCMO films, grown directly on STO and LAO substrates, display out-of-plane c-lattice parameters of c_STO_ = 0.3796 nm and c_LAO_ = 0.3948 nm, indicating a coherent tensile ε_STO_ = 1.7% and compressive ε_LAO_ = − 2.2% stress, respectively. In contrast, the LPCMO film heteroepitaxially grown on MgO possesses a c-lattice parameter of c_MgO_ = 0.3863 nm (see Fig. SM-2c, ref.^41^), which is very close to the bulk LPCMO^3,9^, indicating a stress-free state in agreement with earlier observations^19^.

**Figure 1:**
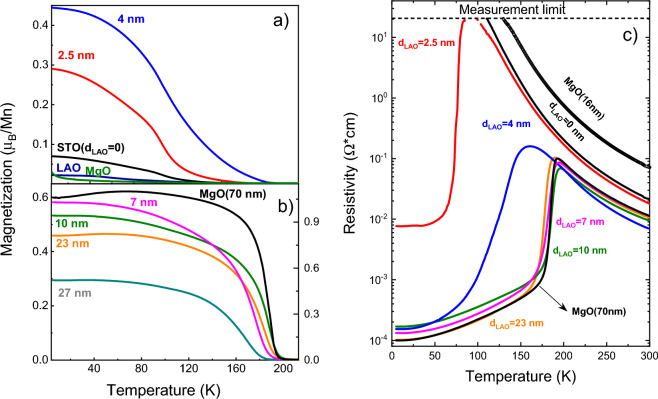
Temperature dependences of: magnetic moment (**a**, **b**) and electrical resistivity (**c**) for thin LPCMO films on STO, LAO and MgO substrates as well as for LPCMO(20 nm)/LAO(d)/STO(100) films with different thickness of LAO buffer layer, d_LAO_ = 0–27 nm. In (**c**) the points between 80 and 100 K for d_LAO_ = 2.5 nm sample are missing because the resistivity exceeds the measurement limit 2*10^1^ Ω*cm.

In Fig. 1 we present the temperature dependences of magnetization (1a), 1b)) and resistivity (1c)) for thin LPCMO films grown on different substrates. One can see that the strained films on STO and LAO, independent of the sign of strain, are weakly magnetic and show insulating behavior. This is in clear contrast with the known bulk behavior of LPCMO with coupled ferromagnetic and metal–insulator transitions at T_C_ = T_MI_ ≈ 200 K^19^. Interestingly, the 16 nm thick film on MgO, being in the overall stress-free state, shows a similar insulating behavior and totally suppressed magnetism. In Fig. 1a one can see only a weak paramagnetic contribution most likely originated from magnetic impurities in the MgO substrate. Remarkably, the bulk-like ferromagnetic metallic behavior has been recovered in a 20 nm thick LPCMO film by introducing a 10 nm thick LAO buffer layer. The bulk-like behavior can also be achieved in an LPCMO/MgO film by increasing its thickness up to 70 nm. In both cases (see Fig. 1) distinct coupled phase transitions can be seen both in the M(T) and ρ(T) curves at a temperature T_C_ = T_MI_ ≈ 194 K. Considering the films on MgO substrate, a recovering of the phase transition and the bulk-like behavior in a strain-free thicker LPCMO film is intimately linked to the reduction of lattice strain and disorder effects caused by misfit dislocations. In other words, the critical thickness of disordered films on MgO measures at least d_0_ ~ 16 nm. Hence, the observation of bulk-like magnetotransport behavior in 20 nm thick LPCMO/LAO/STO film hints to its stress-free state as well as to the absence/diminishing of the disorder at the LPCMO/LAO interface.

To study the influence of the LAO buffer thickness on the elastic decoupling between the LPCMO film and STO substrate we have systematically changed the LAO thickness in the range of d_LAO_ = 0–27 nm. The corresponding results of temperature-dependent magnetic and resistive measurements are also shown in Fig. 1. One can see in Fig. 1a and b that the introduction of an merely 2.5 nm thick LAO buffer drastically changes the magnetotransport: an FM transition with T_C_ = 175 K as well as an MI-like transition with T_MI_ ≈ 80–90 K (see Fig. 1b) were detected. By further increasing the LAO thickness both T_C_ and T_MI_ were found to progressively increase and for d_LAO_ ≥ 7 nm a bulk-like LPCMO magnetotransport has been obtained. Interestingly, for 0 < d_LAO_ ≤ 7 nm the LPCMO films show a complex magnetic behavior with two distinct phase transitions determined from the temperature coefficient of magnetization TCM = 100%(1/M)(dM/dT), shown in Fig. SM-3^41^. Namely, a low T_C1_ = 105–110 K and a high T_C2_ ≈ 136–200 K magnetic phases can be deduced. Note, that T_C1_, being almost constant and close to the temperature of structural phase transition in STO at 105 K^42^, likely indicates the presence of an interfacial magnetic phase at the LPCMO/LAO interface; it may still be elastically coupled to the STO substrate for very thin (d_LAO_ < 7 nm) LAO buffer layers. The Curie temperature T_C2_ increases progressively with increasing the LAO thickness in the range of 0 < d_LAO_ < 7 nm and then saturates for 7 ≤ d_LAO_ ≤ 23 nm, displaying values close to the Curie temperatures of bulk LPCMO T_C_ = 195–200 K. The electrical resistivity behaves similarly (see Fig. 1c) showing ρ(T) curves with a pronounced MI transition at T_MI_ = 190–200 K ≈ T_C_ and relatively low residual resistivity ρ(5K) ≈ 10^–4^ Ωcm for 20 nm thick LPCMO films grown on LAO buffer with thicknesses 7 ≤ d_LAO_ ≤ 23 nm.

**Figure 2:**
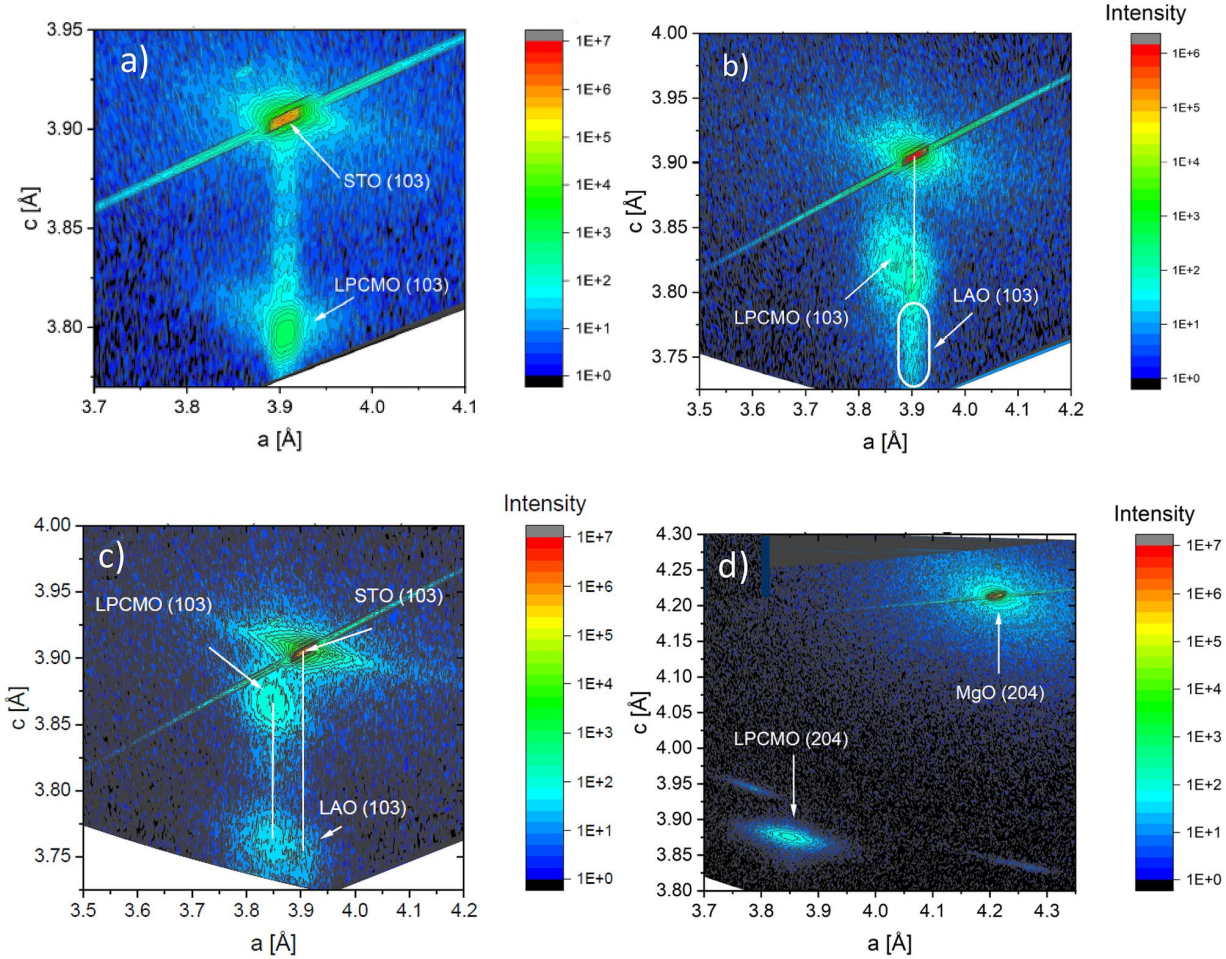
Reciprocal space mapping of: (**a**) LPCMO(30nm)/STO(100); (**b**) LPCMO(20 nm)/LAO(4 nm)/STO; (**c**) LPCMO(20nm)/LAO(10 nm)/STO and (**d**) LPCMO(70nm)/MgO films.

To quantify the strain effect in thin LPCMO films we studied the in-plane and out-of-plane epitaxy of the grown LPCMO/LAO(d_LAO_)/STO heterostructures using reciprocal space mapping (RSM) and X-ray diffraction (XRD), respectively. In Fig. 2a,b and c the RSM patterns of three representative samples with d_LAO_ = 0, 4 and 10 nm are presented, respectively. The RSM of a strain-free 70 nm thick LPCMO/MgO film is also shown in Fig. 2c for comparison. One can see that the LPCMO/STO film (d_LAO_ = 0) is coherently strained as LPCMO and STO possess the same in-plane lattice parameter, a_LPCMO_ = a_STO_ = 0.3905 nm. Correspondingly, the out-of-plane c-lattice parameter, estimated from both RSM (Fig. 2a) and XRD (see Fig. SM-2a, ref.^41^) is significantly reduced: c_LPCMO_ ≈ 0.380 nm. For the very thin buffer layer d_LAO_ = 4 nm (see Fig. 2b) a relaxation of the LPCMO film, accompanied by an increase of its out-of-plane lattice parameter up to c_LPCMO_ ≈ 0.382 nm can be recognized. However, it remains smaller than the pseudo-cubic lattice parameter of the completely relaxed LPCMO/MgO film, c = 0.3863 nm, observed in the corresponding RSM (see Fig. 2d) and XRD (Fig. SM-2b) patterns. The intensity of the XRD peak of the 4 nm thick LAO buffer is too weak to obtaine a reliable quantitative estimate, but nevertheless a tendency to the LAO relaxation can be deduced from the RSM pattern in Fig. 2b. Finally, in the sample with d_LAO_ = 10 nm (Fig. 2c) the LPCMO shows in- and out-of-plane lattice parameters which are very close to the corresponding bulk values, i.e., c_LPCMO_ ~ a_LPCMO_ ≈ 0.386 nm^9^. Moreover, the LPCMO is epitaxially grown on the LAO buffer as they both share almost the same in-plane lattice constant. In addition, the LAO buffer appears to be tetragonally distorted since its out-of-plane parameter c_LAO_ = 0.377 nm is still smaller and the in-plane parameter is significantly larger a_LAO_ ~ 0.385 nm than the bulk value c_LAO_ = 0.379 nm.

An interesting observation can be made in Fig. SM-2e,f^41^, where the XRD patterns for the LAO/STO buffer film have been measured before and after LPCMO deposition. One can see that the c-axis lattice constant of LAO buffer layers with thickness d_LAO_ = 10 and 20 nm shows an increase from c ~ 0.375 and 0.376 nm up to c ~ 0.377 nm after deposition of 20 nm thick LPCMO film. This indicates that the mechanism underlying the elastic film-buffer interaction includes a mutual adjustment of LAO and LPCMO lattices at the heteroepitaxial LPCMO/LAO interface thereby reducing its elastic energy. Being a general property of all epitaxial film/substrate interfaces, such adjustment leading to the increase of the c-lattice parameter of the LAO buffer becomes clearly visible since both LAO buffer and LPCMO film are of comparable thicknesses ~ 10–20 nm. Thus, we can draw two important conclusions: (1) thin LPCMO films are heteroepitaxially grown on the LAO/STO films and (2) the stress in the LPCMO films is controlled by the thickness of the LAO buffer, yielding almost complete stress relaxation for 7 < d_LAO_ < 20 nm.

**Figure 3:**
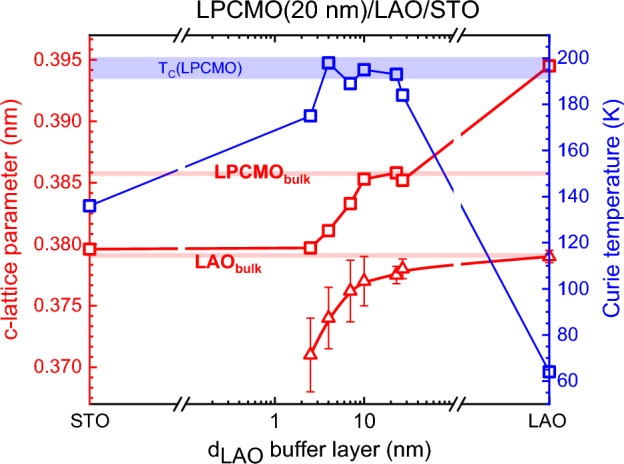
Correlation between the c-lattice parameters (red squares) and Curie temperatures of 20 nm thick LPCMO films grown on the LAO buffer layers versus logarithmic thickness in the range d_LAO_ = 2.5 ÷ 27 nm as well as grown directly on the STO(100) and LAO(100) substrates. C-lattice parameters of the LAO buffers/substrate are shown by red triangles.

In Fig. 3 we summarized the RSM/XRD results and the measured T_C_ values of LPCMO for all samples with different thicknesses of the LAO buffer layers studied here. One can see that for the coherently tensile strained (ε = − 1.7%) film on STO substrate (d_LAO_ = 0 nm) and the compressively strained film on LAO substrate (d_LAO_ → ∞) with ε =  + 2.2% both ferromagnetism and metallicity are suppressed and the ground state of LPCMO seems to be FM insulating with T_C_(STO) ≈ 136 K and T_C_(LAO) ≈ 64 K. The thickness of the LAO buffer does play a decisive role in controlling magnetotransport and leads to a bulk-like ferromagnetic metallic ground state with T_C_ ~ T_MI_ = 190–200 K for nearly relaxed LPCMO films grown on LAO buffer with thickness d_LAO_ = 7–23 nm. For the thicker buffer layer (d_LAO_ = 27 nm) its out-of-plane lattice constant (c_LAO_ ~ 0.3782 nm) approaches the bulk value of the LAO and T_C_ of the LPCMO film decreases down to 184 K (see also Fig. 1b).

**Figure 4:**
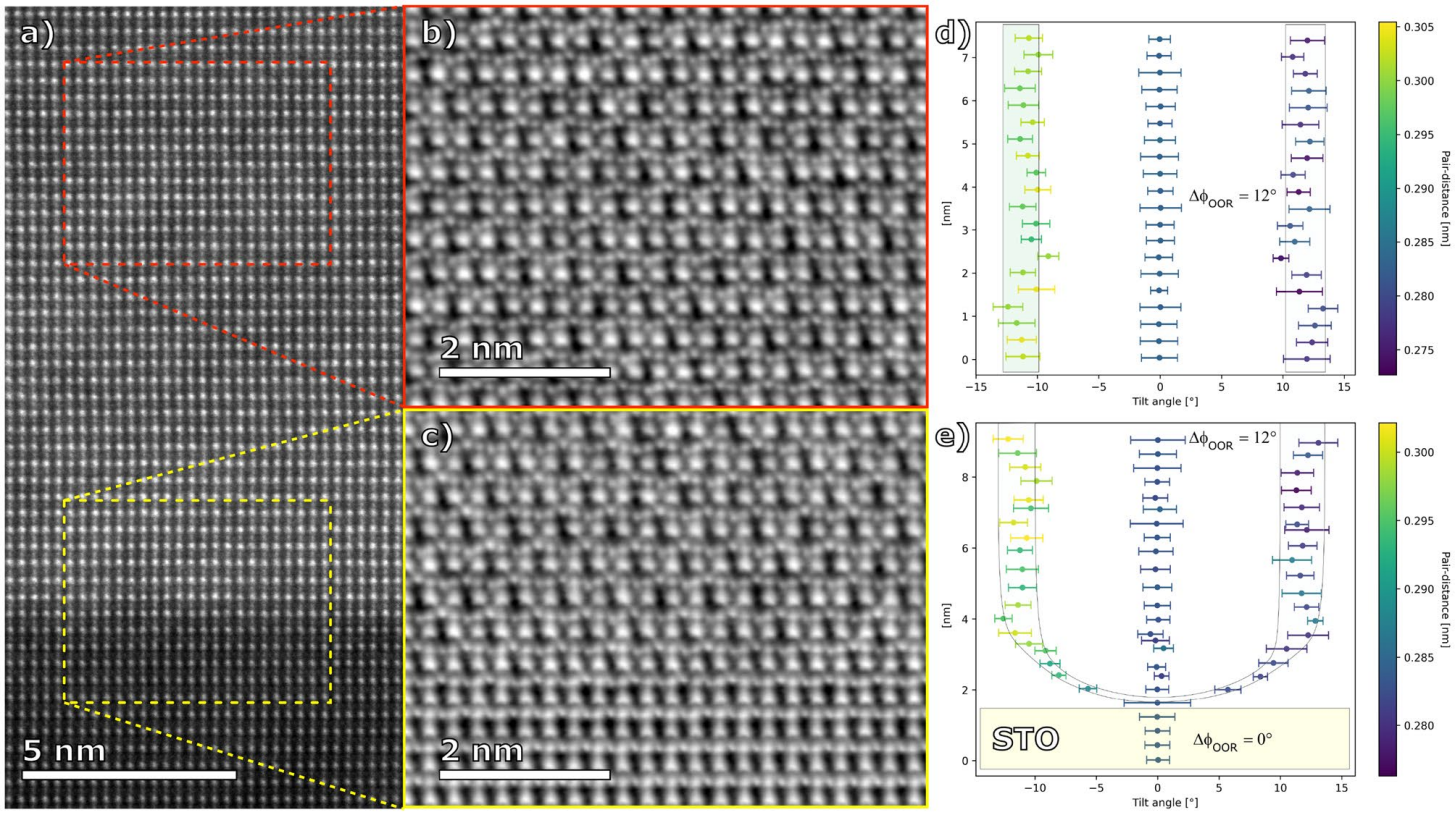
HAADF STEM (**a**) and iDPC-STEM (**b**, **c**) images of LPCMO films grown directly on STO substrate and corresponding statistical distribution evaluation of the oxygen octahedral rotation angles Δφ_OOR_ (**d**, **e**) within each layer along the growth direction.

**Figure 5:**
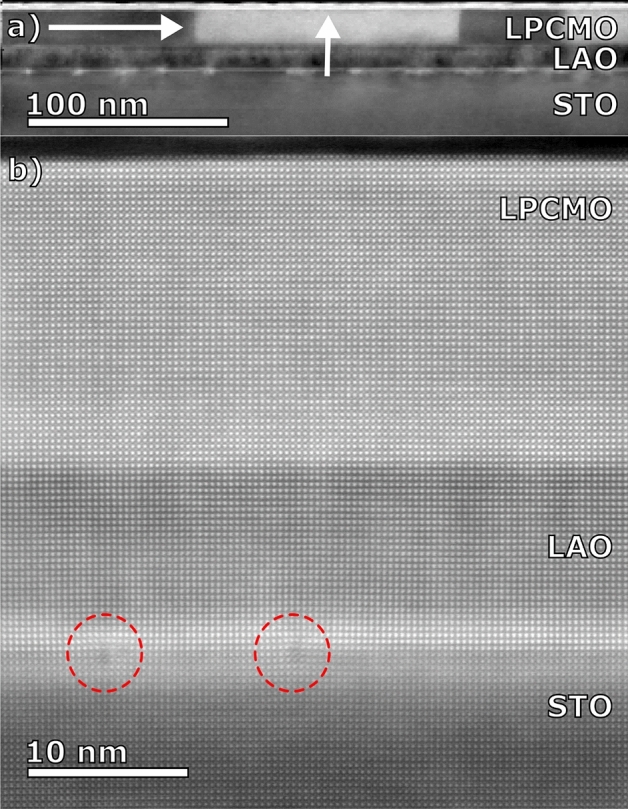
STEM evaluation of LPCMO(20 nm)/LAO(10 nm)/STO(100. (**a**) Orientation mapping of the LPCMO crystallographic b-axis by 4DSTEM nano-diffraction analysis, recorded in STO [100] zone axis orientation; (**b**) ADF-STEM image of the same sample in STO [100] orientation, where edge-type misfit dislocations between STO and LAO are visible when the dislocation line is parallel to the viewing direction, as highlighted by the dashed circles.

We have applied HAADF-STEM and iDPC-TEM techniques with atomic resolution in order to analyze the structure of the LPCMO films and relevant interfaces with the main aim of searching for a structural control parameter responsible for the very different magnetotransport behavior in the strained (on STO) and relaxed (on LAO/STO) LPCMO films. As one can see in Figs. 4 and 5 the respective LPCMO/STO and LPCMO/LAO/STO interfaces are atomically smooth and flat, evidencing that the LPCMO films have been coherently heteroepitaxially grown on the STO and on the LAO/STO substrates. On STO a homogeneous microstructure of a tetragonally distorted LPCMO film due to coherent tensile stress is present (see Fig. 4a). In contrast, the relaxed LPCMO/LAO/STO film reveals twinning domains seen in Fig. 5a with a lateral size of ≈ 30–80 nm. Bright regions represent LPCMO domains where the coherent scattering intensity indicates the b-axis oriented out-of-plane of the thin film, while dark regions correspond to LPCMO domains with b-axis in in-plane direction (either within the image plane or along the projection direction). This kind of microstructure has been previously observed for relatively thick ≈70 nm and relaxed LPCMO/MgO films with bulk-like magnetotransport indicating their orthorhombic Pnma structure^19^. Moreover, at the LAO/STO interface (Fig. 5a and b) one can clearly see an array of misfit dislocations resulting in a partial relaxation of the LAO buffer. The dislocations are irregularly spaced since strain relaxation may also occur along the perpendicular in-plane direction or via mixed defects in the case of a surface step on the STO. As a result, the in-plane lattice constant of the 10 nm thick LAO at the LPCMO/LAO interfaces decreases down to a = 0.385–0.386 nm (see Fig. 2c), thus, yielding a perfect lattice match to the LPCMO film.

**Figure 6:**
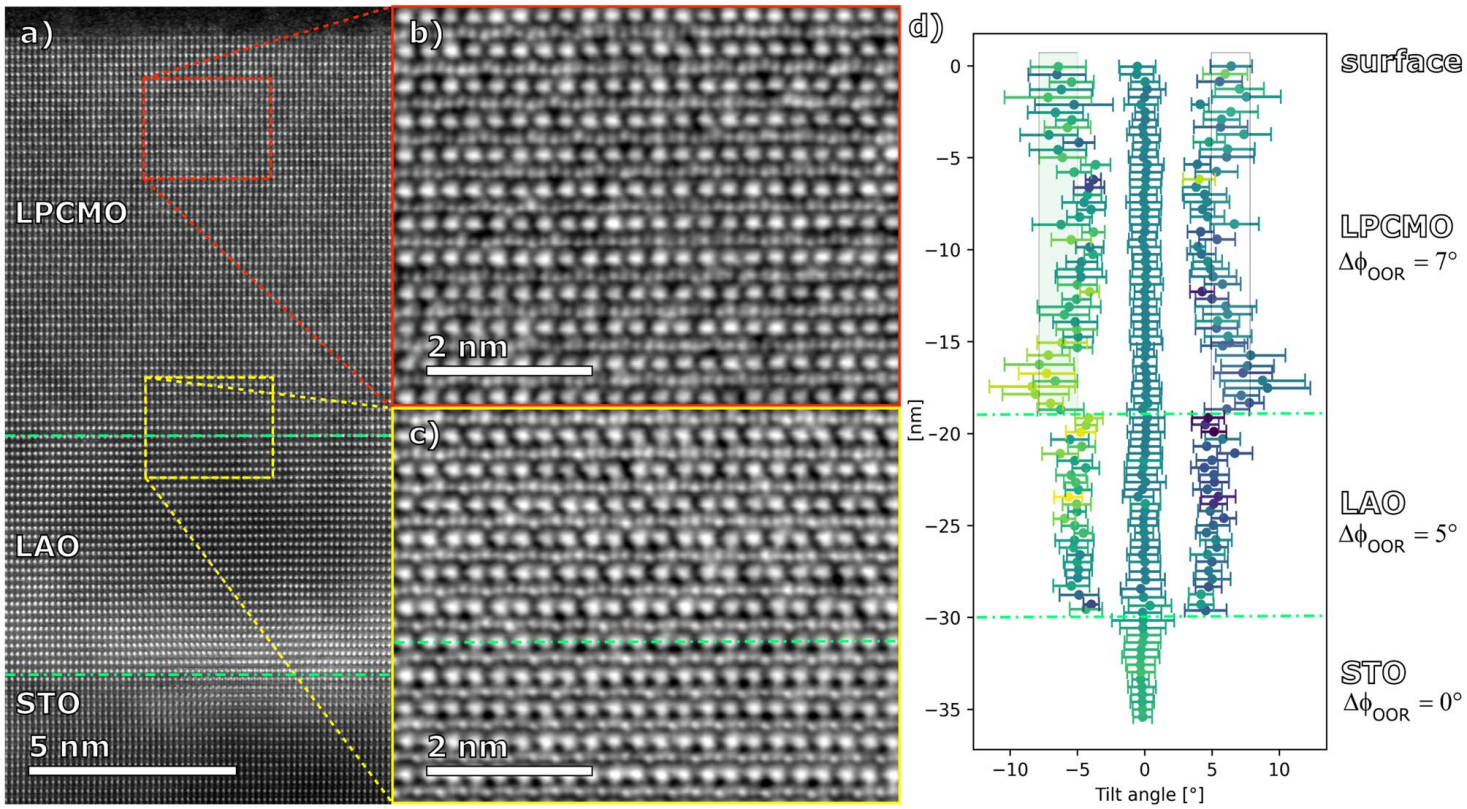
(**a**) HAADF-STEM overview in STO [110] zone axis. Corresponding iDPC-STEM data from selected regions within the LPCMO layer (**b**) and at the LPCMO-LAO interface (**c**). Quantification of Δφ_OOR_ across the entire layer stack (**d**).

A remarkable difference between the tensile strained and relaxed LPCMO films on STO and LAO/STO substrates, respectively, was observed in iDPC-STEM images taken along the [110] zone axis as also shown in Figs. 4 and 6. The iDPC-STEM technique allows visualization of oxygen atoms^43^, and thus to evaluate the Mn–O–Mn tilt/rotation angle, φ_OOR_, which is known to play a decisive role in controlling magnetism and electron transport in perovskite manganites^10^. Note that, the aberration-corrected iDPC technique is able to determine oxygen positions with a pm precision.^44^ The (Mn–O–Mn) bond angles have been evaluated within the plane of zone axis projection in terms of statistics of the bond angle deviation Δφ = 0.5*(180° − φ_OOR_), resolved as a function of the atomic layer number counted along the growth direction. The developed procedure is described in detail in SM^41^ and histograms, obtained from the selected atomic layers within LPCMO films on STO and LAO/STO substrates are shown in Figs. 4d,e and 6d, respectively. One can see in Fig. 4 that an iDPC image of LPCMO/STO film reveals a homogeneous distribution of the Mn–O–Mn angle deviations within the volume of the LPCMO film (see Fig. 4b and d) with Δφ_OOR_ ≈ 12° ± 1.5°, from which an octahedral rotation/tilt angle φ_OOR_ ≈ 156° was obtained. Moreover, within the first 5–6 u.c of the LPCMO close to the LPCMO/STO interface the angle deviation smoothly changes from Δφ = 0° characteristic for the cubic STO to that seen in the body of LPCMO film.

The HAADF-STEM and the corresponding iDPC-STEM images of LPCMO/LAO/STO film shown in Fig. 6 reveal excellent epitaxial growth as well as the change in Δφ_OOR_ within the image projection. Moreover, they indicate that the STO-LAO interface is strained, and the oxygen rotation angle within the LPCMO layer also varies depending on the distance from the surface or LAO-LPCMO interface (see Fig. 6d). The LPCMO film is quantified by significantly smaller Mn–O–Mn angle deviations Δφ ≈ 7° ± 2° and by the correspondingly larger octahedral tilt/rotation angle φ_OOR_ ≈ 166°. However, the average angle deviations Δφ are slightly larger at the LAO-LPCMO interface, while also being more disordered as indicated by the error bars (standard deviation of measurements within one cluster of data points). Note that, due to the presence of twining domains within the LPCMO/LAO/STO film the characteristic zig-zag pattern of φ_OOR_ can only be seen within one domain. Remarkably, both “strained” LPCMO/STO (Fig. 4) and “relaxed” LPCMO/LAO/STO (Fig. 6) interfaces appear sharp not only in the HAADF-STEM but also in the iDPC images. Indeed, the φ_OOR_ angle changes almost abruptly (within 1 u.c.) in case of the “relaxed” LPCMO/LAO/STO interface and within 5–6 u.c. for the “strained” LPCMO/STO one. This is in clear contrast to a much smoother behaviour observed at the La_0.7_Sr_0.3_MnO_3_/STO interfaces in the films grown by pulsed laser deposition^45^: here the Δφ acquires its natural value of ~ 7° or φ_OOR_ ≈ 166° after overcoming a transition zone with a thickness of about 15 u.c.

## Discussion

A drastic difference in the magnetotransport of strained LPCMO/STO(100) and strain-engineered relaxed LPCMO/LAO/STO films has been observed. Namely, the former are insulating and poorly magnetic, whereas the latter possess ferromagnetic metallic ground state similar to that in bulk LPCMO. Furthermore, the smaller and larger octahedral tilt/rotation angles, i.e. φ_OOR_ = 156° and 166°, were determined in strained and relaxed films, respectively. The reason for such differences seems to be a strong tetragonal lattice distortion in the tensile strained LPCMO/STO and its absence in the strain-engineered relaxed LPCMO/LAO/STO. It is likely that tetragonal distortion, reducing the c-lattice parameter down to c = 0.3796 nm (see Fig. SM-1, ref.^41^) and keeping in-plane a- and b-lattice parameters of LPCMO equal to that of the STO due to epitaxy (see Fig. 2), causes a strong decrease of the rotation/tilt angle down to a value even smaller than in the bulk LPCMO.

According to classical neutron diffraction study by Radaelli et al.^10^, the optimally doped A-site substituted bulk manganites with general formula (A_1−y_A’_y_)_0.7_A’’_0.3_MnO_3_ (A, A’ = La, Pr; A’’ = Ca, Sr, Ba) possess different φ_OOR_ (or Mn–O–Mn) angles controlled by the average radius of the A-site cation $\left\langle {{\text{R}}_{{\text{A}}} } \right\rangle$. The φ_OOR_ increases with increasing $\left\langle {{\text{R}}_{{\text{A}}} } \right\rangle$ reflecting the tendency to change crystal structure from the orthorhombic (Pnma) structure for small radii $\left\langle {{\text{R}}_{{\text{A}}} } \right\rangle$ ≤ 0.124 nm and small Mn–O-Mn angles 156° < φ_OOR_ < 164° to the rhombohedral (R-3c) one for larger $\left\langle {{\text{R}}_{{\text{A}}} } \right\rangle$ > 0.124 nm and larger angles 165° < φ_OOR_ < 171°. The here studied LPCMO material in the bulk form with small $\left\langle {{\text{R}}_{{\text{A}}} } \right\rangle$ = 0.1195 possesses small φ_OOR_ = 159° and belongs to an orthorhombic structure^10^. Thus, the observed reduction of the Mn–O–Mn angle for strained LPCMO/STO film down to φ_OOR_ ≈ 156°, which is even smaller than that in bulk LPCMO, is a result of tensile strain and tetragonal distortion. Note that a similarly small φ_OOR_ ≈ 156° has been determined^10^ for bulk Pr_0.7_Ca_0.3_MnO_3_ (PCMO) with a smaller $\left\langle {{\text{R}}_{{\text{A}}} } \right\rangle$ = 0.11793 nm, the ground state of which is known to be an antiferromagnetic insulator^46^. A close similarity with the observed insulating and weakly magnetic ground state of the coherently strained LPCMO/STO film is worth to note. Taken all together this nicely demonstrates correlations, on one hand, between the structure/chemical pressure and magnetism, and, on the other hand, between the epitaxy pressure (stress) and a unique structural parameter φ_OOR_ in the LPCMO films.

Moreover, we have experimentally determined that the mechanism underlying strain relaxation of the LAO buffer grown on STO substrate, having large lattice misfit of 3%, is an abrupt octahedral decoupling at the LAO/STO interface (see Fig. 6) favored by formation of misfit dislocations (see Fig. 5). As a result, the octahedral tilt/rotation angle in the LAO decreases and the in-plane lattice parameter of the partially relaxed LAO increases. Thus, for the thickness d_LAO_ = 7–10 nm one obtains a perfect lattice and angle matching to the LPCMO. Together with the mutual LPCMO/LAO elastic adjustment at the interface, as both layers are of comparable thickness of 10–20 nm, this provides a perfect LPCMO/LAO epitaxy. The observed large φ_OOR_ = 166° in the relaxed LPCMO/LAO/STO film is inherited from the rhombohedral LAO buffer layer which also shows large φ_OOR_ ≈ 170° (see Fig. 5f) close to the φ_OOR_ ≈ 169° in bulk LAO^42^. Note, that the relaxed LPCMO/LAO/STO film still remains in its natural orthorhombic structure as indicated by the twinning domains in the HAADF-STEM (see Fig. 5a) which originate from different a- and b-lattice parameters inherent to the orthorhombic structure. The same twinning domain structure has been observed earlier in the relaxed LPCMO/MgO films^19^.

The improvement of magnetotransport in very thin relaxed LPCMO films is interpreted within the well-known orbital mechanisms controlling magnetism in manganites^47^. First, *orbital disorder*, i.e. no preferred orbital occupation of Mn(3d) orbitals in the relaxed film, as the tetragonal distortion is missing. Second, improved/enhanced Mn(3d)–O(2p) *orbital hybridization* as the Mn–O–Mn bonds are significantly straightened. Third, the resulting enhancement of *double exchange interaction*^48–50^ stabilizes ferromagnetic metallic ground state of the LPCMO/LAO/STO thin films. The fundamentally important structural control parameter^20^, i.e. octahedral tilt/rotation angle was found to be surprisingly large (φ_OOR_ = 166°) in the strain-engineered and relaxed LPCMO/LAO/STO film. In contrast, the strong tetragonal distortion in the strained LPCMO/STO with small φ_OOR_ = 156° leads to an orbital reconstruction with 3dMn_(x2−y2)_ orbitals being more populated^51^ and an enhancement of antiferromagnetic superexchange interaction, which as well favors insulating behavior^47,52^. Thus, we believe our findings could open a promising and relatively simple way to control the colossal magnetoresistive LPCMO films in a wide range of properties by strain-engineered control of octahedral tilt/rotation angle. Namely, by tuning the thickness of the LAO buffer on STO substrate one creates the opportunity not only to grow the relaxed films with bulk-like properties for d_LAO_ = 10–20 nm, but also the tensile (0 < d_LAO_ < 7 nm) and compressively (d_LAO_ > 30 nm) strained films. Such LPCMO films with controllable strain state could be of interest for further studies of CMR physics in perovskite manganites.

## Conclusions

Strain-engineering of thin hetero-epitaxial LPCMO films beyond substrate limitations has been realized within a metalorganic aerosol deposition technique by tuning the thickness of the LAO-buffer on STO(100) substrates. For 7–20 nm thick LAO buffer layers the strain-free LPCMO films with thickness d = 10–20 nm and bulk magnetotransport properties have been obtained. The octahedral tilt/rotation Mn–O–Mn angles, determined by iDPC-STEM, were shown to be very sensitive to the epitaxy strain as a tensile strained LPCMO/STO film shows a reduced φ_OOR_ ≈ 156°. This is shown to be the main reason of the suppressed ferromagnetism and insulating behaviour for strained LPCMO films. A significantly increased φ_OOR_ ≈ 166° for the strain-free LPCMO/LAO/STO film results in an enhancement of double exchange interaction, yielding the optimized ferromagnetic metallic behaviour.

### Supplementary Information

Supplementary Information.

## Data Availability

The datasets used and/or analyzed during the current study available from the corresponding author on reasonable request.
