# Supplementary material for: Tailoring of magnetism & electron transport of manganate thin films by controlling the Mn–O–Mn bond angles via strain engineering
Source: Sci Rep. 2024 Feb 8;14:3253. doi: 10.1038/s41598-024-53722-9 (PMC10853165; doi:10.1038/s41598-024-53722-9)
Supplement: Supplementary file 1 — Supplementary Information. [file 41598_2024_53722_MOESM1_ESM.pdf]

Supplemental Material

Tailoring of Magnetism & Electron Transport of Manganate Thin Films by Controlling the Mn-O-Mn Bond Angles via Strain Engineering

P. Henning[1], R. Gruhl[2], U. Ross[3], V. Roddatis[4], V. Bruchmann-Bamberg[1], K.P. Stroh[1], M. Seibt[3], P. Gegenwart[2] and V. Moshnyaga[1]

[1]Erstes Physikalisches Institut, Georg-August-Universität Göttingen, Friedrich-Hund-Platz 1, 37077 Göttingen, Germany

[2]Experimentalphysik VI, Center for Electronic Correlations and Magnetism, Augsburg University, D-86159 Göttingen, Germany

[3]4th Institute of Physics – Solids and Nanostructures, Georg-August-Universität Göttingen, Friedrich-Hund-Platz 1, 37077 Göttingen, Germany

[4]GFZ German Research Centre for Geosciences, Helmholtz Centre Potsdam, Telegrafenberg, 14473 Potsdam, Germany

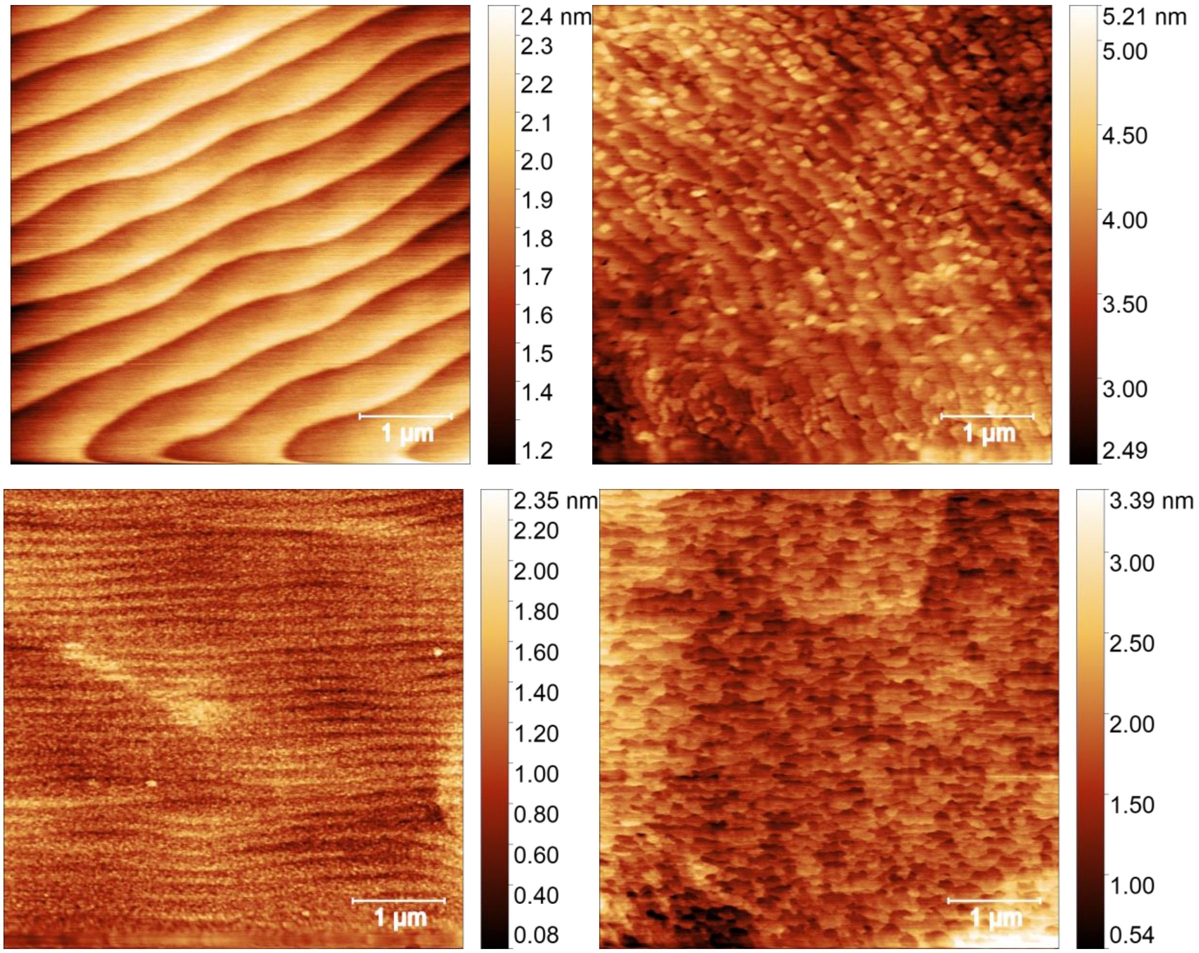

Fig. SM-1 AFM images of a TiO_2-terminated STO(100) substrate (top, left), LPCMO(30 nm)/STO(100) film (top, right), LAO(10 nm)/STO(100) (bottom, left) and LPCMO(20 nm)/LAO(10 nm)/STO(100) film (bottom, right).

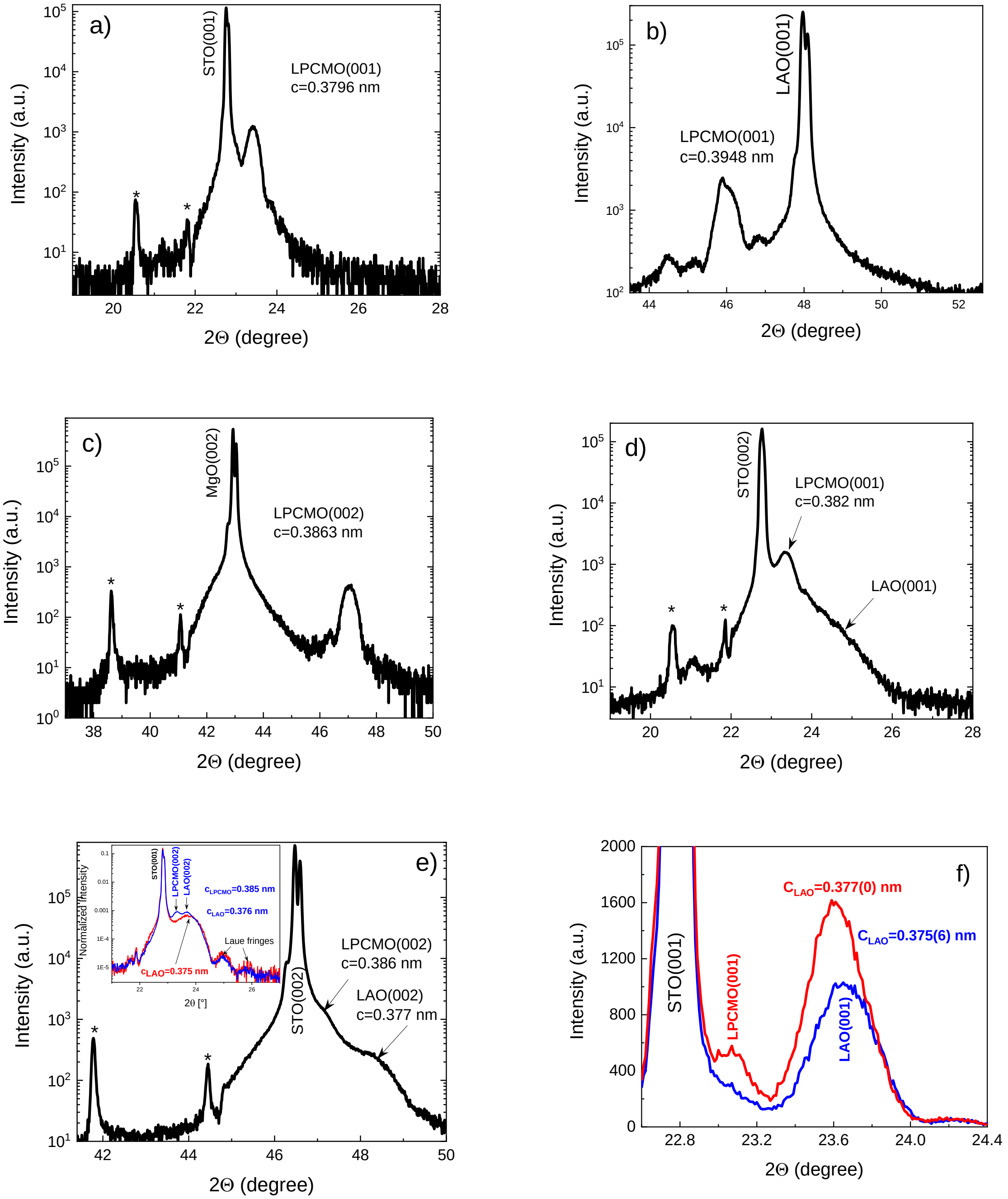

Fig. SM-2 XRD patterns of the representative films in the vicinity of substrate (001) or (002) peaks: a) tensile strained 30 nm thick LPCMO/STO film; b) compressively strained 16 nm thick LPCMO/LAO(100); c) stress-free 20 nm thick LCMO/MgO(200); d) tensile strained LPCMO(20nm)/LAO(4nm)/STO; e) strain-free LPCMO(20nm)/LAO(10nm)/STO and f) strain-free LPCMO(20nm)/LAO(22nm)/STO film with thickness. The inset in e) as well as Fig. SM-2f) show the increase of the XRD intensity and the c-lattice parameter of the LAO buffer layer after deposition of the LCPMO film.

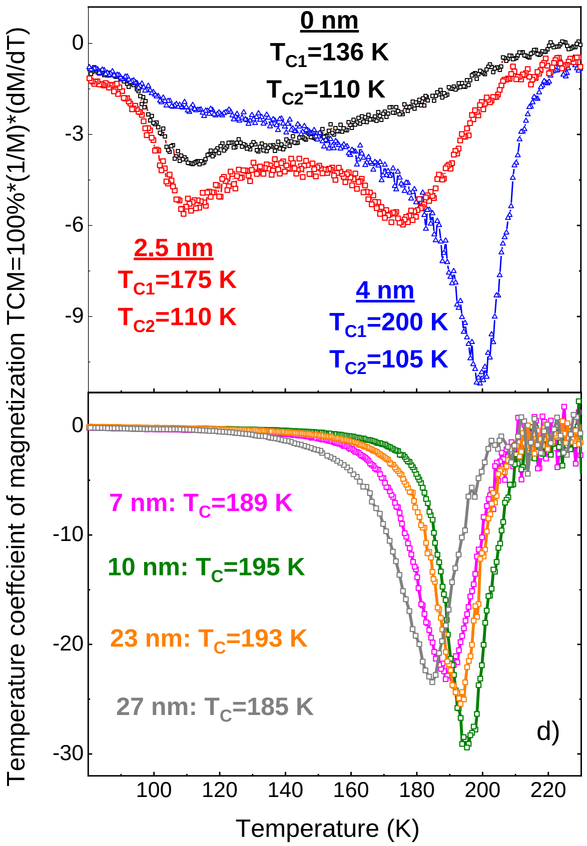

Fig. SM-3 Temperature coefficient of magnetization as a function of temperature for LPCMO(20nm)/LAO(d)/STO(100) films with different thickness of LAO buffer layer, d_{LAO}=0-27 nm

Evaluation procedure of octahedral tilt angles

The octahedral tilt from atomic-resolution iDPC STEM images was evaluated using the Atomap library [*M. Nord et al, Advanced Structural and Chemical Imaging 2017 3:9* https://doi.org/10.1186/s40679-017-0042-5] for Python in order to find the positions of atomic column contrast and classify them into either A-site cation (La/Pr/Ca/...) or Mn-O sublattice. Subsequently, positions were refined by fitting of 2D Gaussians. The array of all atomic column positions was evaluated regarding all nearest and next-nearest neighbour vectors within the image plane. Therefore, the nearest-neighbour vectors correspond to the Mn-O bonds projected onto the zone axis, and the second-nearest neighbour vector within the Mn-O plane corresponds alternatingly to the vector spanning one O-Mn-O octahedron, or the connecting vector between two neighboring Mn columns. The orientation (tilt) of individual vectors vs. the mean orientation within one lattice plane is then plotted against the perpendicular spatial dimension, i.e. the growth direction in the case of octahedral tilt analysis, in order to determine statistical trends.
